# Maternal glycemia in pregnancy is longitudinally associated with blood DNAm variation at the *FSD1L* gene from birth to 5 years of age

**DOI:** 10.1186/s13148-023-01524-7

**Published:** 2023-06-29

**Authors:** Amélie Taschereau, Kathrine Thibeault, Catherine Allard, Diana Juvinao-Quintero, Patrice Perron, Sharon M. Lutz, Luigi Bouchard, Marie-France Hivert

**Affiliations:** 1 Department of Biochemistry and Functional Genomics, Faculty of Medicine and Health Sciences (FMHS), Université de Sherbrooke, Sherbrooke, QC Canada; 2 Centre de Recherche du Centre hospitalier universitaire de Sherbrooke (CR-CHUS), Sherbrooke, QC Canada; 3 Department of Medicine, FMHS, Université de Sherbrooke, Sherbrooke, QC Canada; 4 Department of Population Medicine, Harvard Pilgrim Health Care Institute, Harvard Medical School, Boston, MA USA; 5 Department of Biostatistics, Harvard T.H. Chan School of Public Health, Boston, MA USA; 6 Clinical Department of Laboratory Medicine, Pavillon des Augustines, Centre intégré universitaire de santé et de services sociaux (CIUSSS) du Saguenay-Lac-Saint-Jean – Hôpital de Chicoutimi, 305 rue St-Vallier, Saguenay, QC G7H 5H6 Canada; 7 Diabetes Unit, Massachusetts General Hospital, Boston, MA USA; 8 Department of Epidemiology, Harvard T.H. Chan School of Public Health, Boston, MA USA

**Keywords:** Epigenetics, DNAm, Fetal programming, Gestational hyperglycemia, Pregnancy, Offspring

## Abstract

**Background:**

In utero exposure to maternal hyperglycemia has been associated with an increased risk for the development of chronic diseases in later life. These predispositions may be programmed by fetal DNA methylation (DNAm) changes that persist postnatally. However, although some studies have associated fetal exposure to gestational hyperglycemia with DNAm variations at birth, and metabolic phenotypes in childhood, no study has yet examined how maternal hyperglycemia during pregnancy may be associated with offspring DNAm from birth to five years of age*.*

**Hypothesis:**

Maternal hyperglycemia is associated with variation in offspring DNAm from birth to 5 years of age.

**Methods:**

We estimated maternal hyperglycemia using the area under the curve for glucose (AUC_glu_) following an oral glucose tolerance test conducted at 24–30 weeks of pregnancy. We quantified DNAm levels in cord blood (*n* = 440) and peripheral blood at five years of age (*n* = 293) using the Infinium MethylationEPIC BeadChip (Illumina). Our total sample included 539 unique dyads (mother–child) with 194 dyads having DNAm at both time-points. We first regressed DNAm *M*-values against the cell types and child age for each time-point separately to account for the difference by time of measurement for these variables. We then used a random intercept model from the linear mixed model (LMM) framework to assess the longitudinal association between maternal AUCglu and the repeated measures of residuals of DNAm. We adjusted for the following covariates as fixed effects in the random intercept model: maternal age, gravidity, smoking status, child sex, maternal body mass index (BMI) (measured at first trimester of pregnancy), and a binary variable for time-point.

**Results:**

In utero exposure to higher maternal AUC_glu_ was associated with lower offspring blood DNAm levels at cg00967989 located in *FSD1L* gene (*β* = − 0.0267, *P* = 2.13 × 10^–8^) in adjusted linear regression mixed models. Our study also reports other CpG sites for which DNAm levels were suggestively associated (*P* < 1.0 × 10^–5^) with in utero exposure to gestational hyperglycemia. Two of these (cg12140144 and cg07946633) were found in the promotor region of *PRDM16* gene (*β*: − 0.0251, *P* = 4.37 × 10^–07^ and *β*: − 0.0206, *P* = 2.24 × 10^–06^, respectively).

**Conclusion:**

Maternal hyperglycemia is associated with offspring DNAm longitudinally assessed from birth to 5 years of age.

**Supplementary Information:**

The online version contains supplementary material available at 10.1186/s13148-023-01524-7.

## Background

Gestational diabetes mellitus (GDM) is defined by hyperglycemia first occurring during pregnancy in an individual who did not previously have diabetes [1]. GDM affects approximately 14% of pregnancies worldwide [2] with variation in prevalence depending on ethnicity [3] and the diagnostic criteria applied [4]. Regardless of GDM diagnosis, maternal hyperglycemia along the whole spectrum has been linearly associated with adverse perinatal outcomes in both mother and offspring [5]. Exposure to maternal hyperglycemia in utero is also associated with an increased risk for the development of chronic diseases later in childhood, including obesity [6], metabolic [7] and cardiovascular diseases [8], asthma [9], autism [10], as well as other adverse neurodevelopmental outcomes [11]. The Developmental Origin of Health and Disease (DOHaD) hypothesis is central to the concept of fetal programming and based on lasting fetal metabolic adaptions to environmental/maternal stressors during the critical developmental period [12]. Epigenetic marks are highly malleable during in utero development and in early life but can be stable over time [13]. Epigenetics is one of the most likely molecular mechanisms as part of the DOHAD theory and fetal metabolic programming.

DNA methylation (DNAm) is the most studied epigenetic modification. It involves the addition of a methyl group to the fifth carbon of a cytosine when located just upstream of a guanine [14]. Growing evidence supports that DNAm may be the bridge between prenatal exposure to maternal hyperglycemia and the predisposition to chronic disease in later life. The most comprehensive study consists of a meta-analysis of an epigenome-wide association study (EWAS) investigating associations between maternal hyperglycemia exposure and cord blood DNAm variations. This study reported significant associations (FDR < 0.05) at two loci between cord blood DNAm and the area under the curve of glucose (AUC_glu_) following an oral glucose tolerance test (OGTT) [15]. Candidate gene studies also reported significant correlations between maternal hyperglycemia exposure during pregnancy and offspring DNAm in genes implicated in chronic disease pathways [16, 17].

However, although the association between maternal hyperglycemia exposure and DNAm measured at birth has been explored in a few studies, no study has yet examined how maternal hyperglycemia may be associated with offspring DNAm from birth to five years of age. To address this gap, we conducted an EWAS examining the association between maternal glycemic response to an OGTT performed during pregnancy, and offspring DNAm assessed longitudinally at birth (cord blood) and at age 5 years (peripheral blood), using modeling approaches that integrate DNAm at both time-points.

## Methods

### Study population

Our study sample consists of 539 mother–child dyads from the Genetics of Glucose Regulation in Gestation and Growth (Gen3G) prospective cohort. We recruited Gen3G participants in the Sherbrooke City area, Canada, to investigate environmental and genetic determinants of glucose regulation in pregnancy, and their impacts on fetal development and offspring health [18]. Between 2010 and 2013, we invited pregnant women to participate in the study when they attended a routine prenatal blood sampling visit during their first trimester of pregnancy at the Centre Hospitalier Universitaire de Sherbrooke (CHUS), if they planned to deliver at CHUS [18]. Eligible women were ≥ 18 years old, with a singleton pregnancy, and not reporting using medication that alters glucose. We excluded women with pre-existing diabetes or diabetes diagnosed in the first trimester based on laboratory screening (A1c > 6.5% or 1 h-glucose ≥ 10.3 mmol/L post-50 g glucose challenge test [GCT]). Details on Gen3G participants have been published previously [18]. All women provided signed consent, and the CHUS IRB approved all Gen3G protocols.

At enrollment, women provided information on age, ethnicity, smoking habits during pregnancy (never/former smoker vs current smoker) and gravidity (primigravid vs multiparous). During the first (6–14 weeks) and second (24–30 weeks) pregnancy visits, research staff measured weight (in kg) and height (in m) using standardized procedures, and we calculated body mass index (BMI) using the standard formula (kg/m^2^) [18]. At the first visit, most women completed a non-fasting 50 g-GCT. At the second visit, all women conducted a 75 g-OGTT in order to measure glucose tolerance. At delivery, we abstracted from the medical records information about gestational age at birth (in weeks), child sex, and birth weight (in g). Research staff collected umbilical cord blood samples rapidly after delivery (< 30 min).

We conducted in-person follow-up visits with mother–child pairs at the CHUS Research Center approximately 5 years after birth. During this visit, we collected updated medical history and anthropometric measures, in addition to child blood samples.

### Maternal glucose measured during a 75 g-OGTT in the second trimester, and estimation of the area under the curve for glucose

We measured glucose levels (mmol/L) using the glucose hexokinase method (Roche Diagnostics, Indianapolis, US) at the CHUS biochemistry clinical laboratory rapidly after blood collection [18, 19]. We calculated the AUC_glu_ based on the trapezoidal formula [20], to capture a global measure of maternal hyperglycemia in pregnancy that encompasses all three time-points of the OGTT since all of them have been associated with adverse pregnancy outcomes [5], and the AUC_glu_ was the most informative phenotype in prior EWAS investigating dysregulation in child cord blood DNAm [15].

### Genome-wide DNA methylation level assessment

**Fig. 1:**
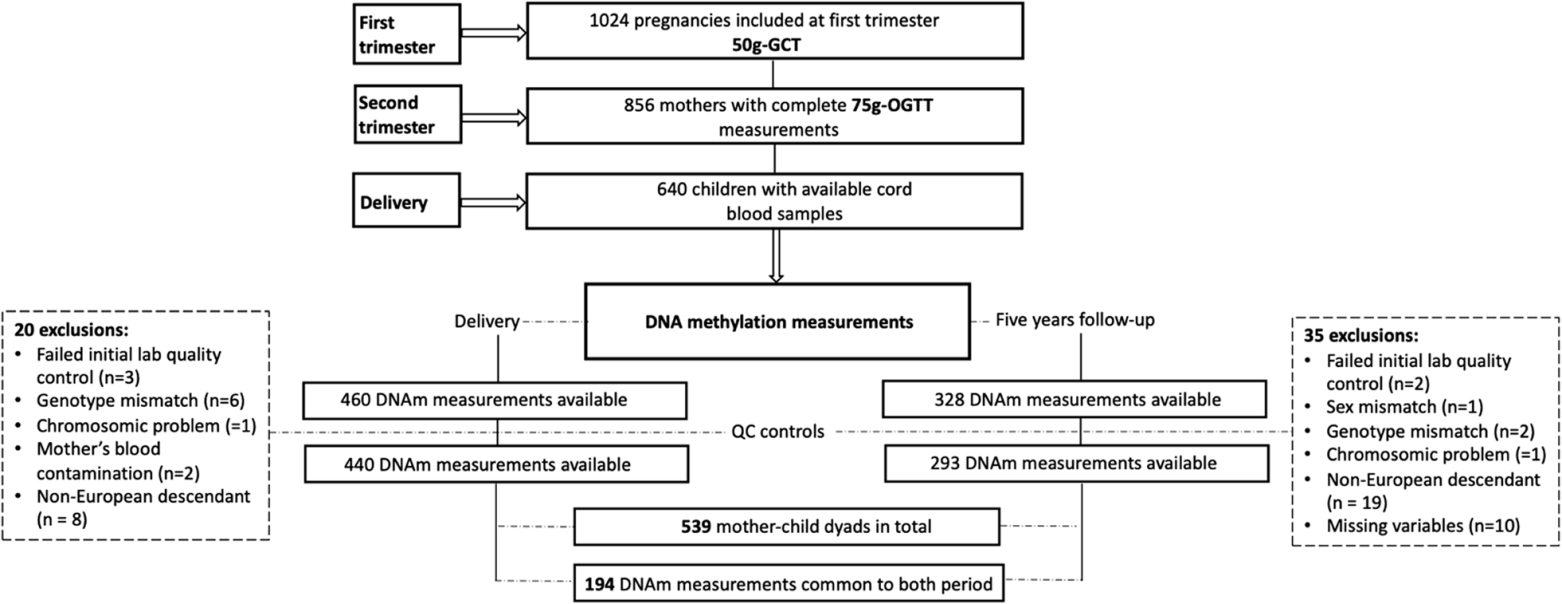
Flowchart illustrating the participant selection from the Gen3G prospective cohort

Genomic DNA was purified from umbilical cord blood cells and from blood cells, respectively, collected at delivery and 5 years of age using the AllPrep DNA/RNA/Protein Mini kit (QIAGEN, Hilden, Germany). DNA was then bisulfite converted with the EZ-Methylation kit (Zymo Research, CA, US), and subsequently analyzed for DNAm at > 850,000 CpG sites at single-nucleotide resolution using the Infinium MethylationEPIC BeadChip (Illumina, San Diego, CA). We first performed a manual inspection of the quality of the methylation assay looking at probes call rate (> 99%), color balance, staining, extension, hybridization, specificity and bisulfite conversion (probe types I and II) using the BeadArray Controls Reporter software. We transferred raw methylation files into R (version 4.1.1) for further preprocessing using the *minfi* R package [21]. Our final dataset included 440 cord blood samples and 293 peripheral blood samples collected at the ~ 5-year visit after quality controls. In terms of participants, this represents 539 unique dyads (mother–child pairs), from which 194 dyads had both time-points (cord blood and peripheral blood) included in the analyses (see support Fig. 1).

We implemented functional normalization for background and dye bias correction [22] of the methylation data, and the regression on correlated probes (RCP) method [23] to adjust for probe-type bias, as previously described [24]. We filtered out probes with null methylation variance and detection *P-*value > 0.05 in at least 5% of the samples. We annotated CpG sites in the array using the R package *IlluminaHumanMethylationEPICmanifest* (version 1.0 B4), and we excluded probes located on sex chromosomes, non-CpG probes (“rs” or “ch”), single-nucleotide polymorphism (SNP)-associated probes at the CpG site, the single-base extension, or anywhere within the probe, if the SNP had a minor allele frequency (MAF) ≥ 0.05. In addition, we removed cross-reactive probes as previously described [25]. We used ComBat [26] to correct for technical variability and batch effects, while protecting our variables of interest. The model thus included the three glucose values (fasting glucose, 1 h-glucose, and 2 h-glucose post-OGTT), maternal age, BMI in the first trimester of pregnancy, gravidity, smoking status, and child sex. After quality control, we included up to 719,360 high-quality CpG sites in the analysis using DNAm from combined cord blood and childhood peripheral blood. We calculated the predicted cell counts in cord blood based on the method described by Gervin et al.[27], and the predicted cell counts in child blood based on the method from Salas et al.[28], implemented in the *FlowSorted.Blood.EPIC* R package.

### Statistical analyses

We described baseline characteristics of the overall sample, and of samples at each time-point, using standard descriptive statistics. For each CpG site, we used methylation values measured in the *β*-value scale, as a range estimating between 0% (unmethylated) and 100% (completely methylated), to transform into *M*-values prior to analyses based on the statistical validity of this method to identify differential DNAm [29]. Due to differences in the cell types predicted in each time-point (i.e., cord blood = CD8 + T cells, CD4 + T cells, monocytes, natural killer, B cells, granulocytes and nucleated red blood cells (nRBC); peripheral blood at ~ 5 years = CD8 + T cells, CD4 + T cells, monocytes, natural killer, B cells and neutrophils) and different age measures (cord blood: gestational age in weeks; peripheral blood: child age in years), we regressed DNAm *M*-values against the cell types and age for each time-point separately, and retrieved residuals from this association to perform the longitudinal EWAS. For the linear mixed model (LMM), we considered a random intercept model using the *nlme* R package [30] to assess the longitudinal association between maternal AUC_glu_ (primary exposure) and repeated measures of residuals of DNAm (in *M*-values adjusted for cell types and age, respectively, in each DNAm time-point) at ~ 719,360 CpG sites as outcomes. We conducted similar analyses for individual glucose components of maternal AUC_glu_: fasting glucose, 1 h-glucose, and 2 h-glucose post-OGTT as secondary exposures. We included the following covariates as fixed effects: maternal age, gravidity (primigravid vs multigravida), smoking in the first trimester of pregnancy (smoker versus non-smoker), child sex, maternal BMI in the first trimester of pregnancy, and a binary variable for time-point (i.e., birth = 0, early childhood = 1). We used a random intercept for the *subject* to account for the correlation of multiple measures on the same individual. We also conducted separate linear models for individual CpG at each of the two time-points (cord blood and 5y peripheral blood) using blood DNAm transformed in the same way (residuals on cell counts and age), for all four maternal glycemic exposures (maternal AUC_glu_, fasting glucose, 1 h post-OGTT and 2 h post-OGTT) using the same covariates (without the time-point variable). We also explored if we could find differentially methylated regions (DMRs) using the DMRff method [31] using the residuals that were used for the EWAS as the individual-level data. We choose this method as it provides decent power for detecting DMRs and without an inflated Type I error rate [32].

We reported coefficient estimates at the individual CpG site as the mean difference in DNAm residuals on cells and age, per unit increase in the AUC_glu_. We calculated the genomic inflation factor or lambda (*λ*) for each EWAS (Additional file 1). We adjusted associations for multiple testing using Bonferroni, deeming epigenome-wide significant associations as *P* < 6.9 × 10^–8^, while we considered “suggestive” associations identified with an arbitrary *P* ≤ 1.0 × 10^–5^. For each top CpG site (*P* ≤ 1.0 × 10^–5^ in at least one model (mixed model, or linear models in cord blood and 5y peripheral blood)), we visually inspected outliers and distribution by plotting the residuals (standardized or not) versus the fitted values, and by using quantile plots. Top CpG sites identified in the EWAS were annotated to their gene using the Illumina annotation (*IlluminaHumanMethylationEPICanno.ilm10b2.hg19* R package [33]) and if not annotated, we completed using the closest gene using the UCSC Gene Browser. We visualized the results using Manhattan and quantile plots. All analyses were performed in R (version 4.1.1).

## Results

### Participants’ characteristics

The characteristics of the mothers and children are shown in Table 1. Briefly, mothers were 28.2 ± 4.2 years old (mean ± SD) and had a median [IQR] BMI of 23.9 [21.6; 27.9] kg/m^2^ at the first trimester of pregnancy (median 9.4 [8.1; 11.7] weeks). Forty-nine (9.1%) women reported smoking and 180 (33.4%) were primigravid. Median and IQR values of maternal glycemia during the 75 g OGTT (median gestational age; 26.3 [25.9; 27.1] weeks) are indicated in Table 1. The median AUC_glu_ was 12.0 [10.5; 13.4] mmol/L*h and fifty (9.3%) women developed GDM. At delivery, the median gestational age was 39.7 [38.9; 40.4] weeks and 252 of these children were female (46.8%). At the 5-year visit, the children had a median age of 5.2 [5.1; 5.4] years old and 130 (44.4%) were female.

**Table 1:** Characteristics of Gen3G mother–child pairs during pregnancy, delivery, and child at ~ 5 years

	*N*	Mean ± SD or median [IQR] or *N* (%)
*Maternal measures – 1st trimester*		
Age, years	539	28.2 ± 4.2
Gestational age, weeks	539	9.4 [8.1; 11.7]
Primigravid, yes	539	180 (33.4%)
Smoking status, yes	539	49 (9.1%)
BMI, kg/m^2^	539	23.9 [21.6; 27.9]
*Maternal measures – 2nd trimester*		
Gestational age, weeks	539	26.3 [25.9; 27.1]
GDM, yes	539	50 (9.3%)
Glucose fasting, mmol/L	539	4.2 [3.9; 4.4]
Glucose 1 h post-OGTT, mmol/L	539	7.1 [6.0; 8.2]
Glucose 2 h post-OGTT, mmol/L	539	5.7 [4.8; 6.6]
AUC_glu_, mmol/L * h	539	12.0 [10.5; 13.4]
*Measures at delivery*		
Gestational age, weeks	539	39.7 [38.9; 40.4]
Sex, girls	539	252 (46.8%)
*Child measures – 5 years*		
Age, years	293	5.2 [5.1; 5.4]
Sex, girls	293	130 (44.4%)

*AUC*_*glu*_, Area Under the Curve of glucose; *BMI*, Body Mass Index; *GDM*, Gestational Diabetes Mellitus; *SD*, Standard Deviation

### ***Maternal AUC***_***glu***_*** is associated with DNAm variations in cord blood and in blood at 5 years of age***

**Fig. 2:**
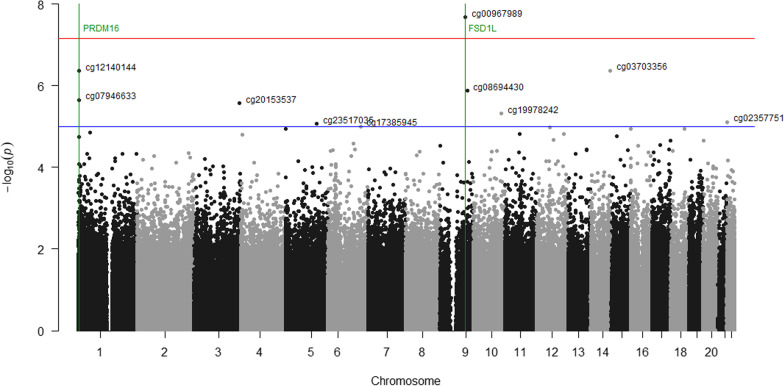
Epigenome-wide association plot (− log10 *P*-values) for maternal AUC_glu_. Manhattan plot for the EWAS of maternal AUC_glu_ following a 75 g-OGTT with DNAm measured longitudinally at birth and five years of age (red line: Bonferroni threshold = *P*-value < 6.9 × 10^–8^; blue line: Suggestive threshold = *P* < 1.0 × 10^–5^)

**Fig. 3:**
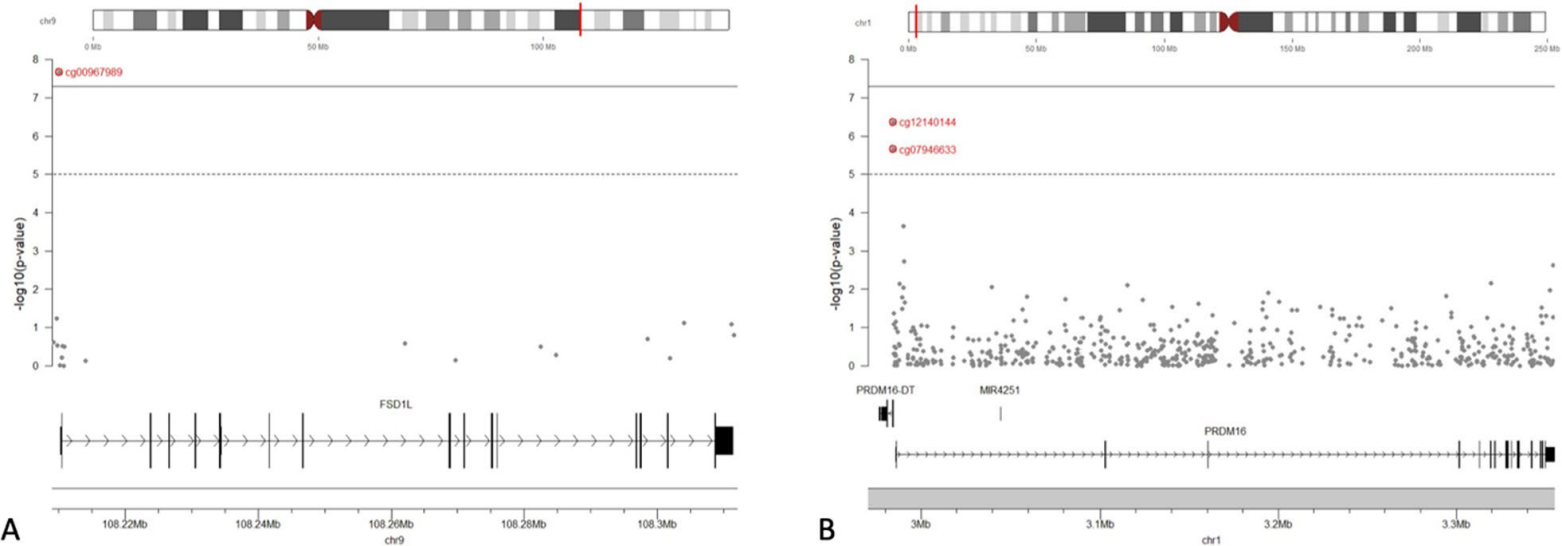
*FSD1L* and *PRDM16* gene regional plot association (− log10 *P*-values) for maternal AUC_glu._ Genomic regional plots for **a** *FSD1L* (on Chr 9) and **b** *PRDM16* (on Chr 1) genes of maternal AUC_glu_ following a 75 g-OGTT with DNAm measured longitudinally at birth and five years of age (Solid line: Bonferroni threshold = *P*-value < 6.9 × 10^–8^; dashed line: Suggestive threshold = *P* < 1.0 × 10^–5^)

We first identified a total of 10 CpG sites (9 loci) at which offspring DNAm levels were associated with exposure to maternal AUC_glu_ at a suggestive threshold of *P*-value ≤ 10^–5^ (Fig. 2). The strongest association reached the genome-wide significant threshold and was observed at the cg00967989 located within the proximal promotor of the *FSD1L* gene (*β* = − 0.0267, *P* = 2.13 × 10^–8^) (Fig. 3a). Among the other loci with suggestive associations, cg07946633 (*β* = − 0.0206, *P* = 2.24 × 10^–6^) and cg12140144 (*β* = − 0.0251, *P* = 4.37 × 10^–7^) were both located within the promotor region of the *PRDM16* gene (Fig. 3b). The seven other CpG sites were located at the *HES1*, *CCDC28A*, *INPP5F*, *CKB, GP1BB*/*SEPT5*, *SAR1B,* and *RGS3* gene loci. We presented in Table 2 the complete list of CpGs at which offspring blood DNAm levels were associated with exposure to high maternal AUC_glu_ at a suggestive threshold (*P* ≤ 1.0 × 10^–5^), their associated genes, genomic location, regression *β* values and *P*-values from the main longitudinal EWAS (based on LMM). Using DMRff, we identified two DMRs, one containing the two CpG sites (cg07946633 and cg12140144; adj *P* = 0.03) previously reported at the *PRDM16* gene locus and the second containing the one CpG site (cg00967989; adj *P* = 0.02), previously reported at the *FSD1L* gene locus (Additional file 2).

**Table 2:** CpG sites identified (suggestive *P* < 10^–5^) in linear mixed models (LMM) testing associations between maternal AUC_glu_ and DNAm measured in cord blood and blood at 5 years of age

CpGs	Chr	Position	Cord blood DNAm levels in *β*-value Mean ± SD *N* = 440	Five years blood DNAm levels in *β*-value Mean ± SD *N* = 293	Nearest gene	LMM regression estimates
cg00967989	**9**	**108,210,147**	**0.013 ± 0.002**	**0.015 ± 0.003**	***FSD1L***	***β*: **− **0.0267** ***P*: 2.13 × 10**^**–08**^
cg03703356	14	103,989,368	0.073 ± 0.012	0.132 ± 0.016	*CKB*	*β*: − 0.0217 *P*: 4.25 × 10^–07^
cg12140144	1	2,984,275	0.017 ± 0.004	0.045 ± 0.008	*PRDM16*	*β*: − 0.0251 *P*: 4.37 × 10^–07^
cg07946633	1	2,984,245	0.251 ± 0.034	0.339 ± 0.032	*PRDM16*	*β*: − 0.0206 *P*: 2.24 × 10^–06^
cg08694430	9	116,420,234	0.864 ± 0.032	0.838 ± 0.035	*RGS3*	*β*: 0.0324 *P*: 1.33 × 10^–06^
cg20153537	3	193,917,170	0.949 ± 0.017	0.944 ± 0.007	*HES1*	*β*: − 0.0223 *P*: 2.70 × 10^–06^
cg19978242	10	121,578,846	0.856 ± 0.030	0.886 ± 0.026	*INPP5F*	*β*: − 0.0322 *P*: 4.85 × 10^–06^
cg02357751	22	19,710,880	0.014 ± 0.003	0.030 ± 0.015	*GP1BB; SEPT5*	*β*: − 0.0368 *P*: 7.71 × 10^–06^
cg23517035	5	133,971,253	0.954 ± 0.010	0.939 ± 0.011	*SAR1B*	*β*: 0.0212 *p*: 8.68 × 10^–06^
cg17385945	6	139,095,355	0.010 ± 0.002	0.014 ± 0.003	*CCDC28A*	*β*: − 0.0257 *p*: 9.93 × 10^–06^

Model adjusted for maternal age, gravidity, smoking status, child sex, BMI at first trimester of pregnancy and the binary variable for time-point. Significant result after Bonferroni correction with *P*-value < 6.9 × 10^–8^ are in bold

*AUC*_*glu*_, Area Under the Curve of glucose; *Chr*, Chromosome; *CpG*, Cytosine–phosphate–Guanine; *DNAm*, DNA methylation; *SD*, Standard deviation

We also tested associations between maternal AUC_glu_ and DNAm measured at birth (cord blood) and at 5 years (peripheral blood), separately (Additional file 3). When looking at individual time-points for *FSD1L*, the signal appears to be primarily driven by its association in childhood, with only nominal association observed at birth. A similar pattern applies to the CpGs localized at the *GP1BB/SEPT5* gene locus. In contrast, the signals identified for *PRDM16* (cg12140144), *SAR1B, CCDC28A,* and *INPP5F* appear to be primarily driven by their associations reported at birth. Interestingly, the signal observed for *PRDM16* (cg07946633) appears to be equally driven by its associations at birth and at age 5 years with a similar pattern in *HES1, CKB,* and *RGS3* genes.

### ***Most of the CpG sites associated with maternal AUC***_***glu***_*** were associated with 1 h and 2 h post-OGTT glucose levels***

**Fig. 4:**
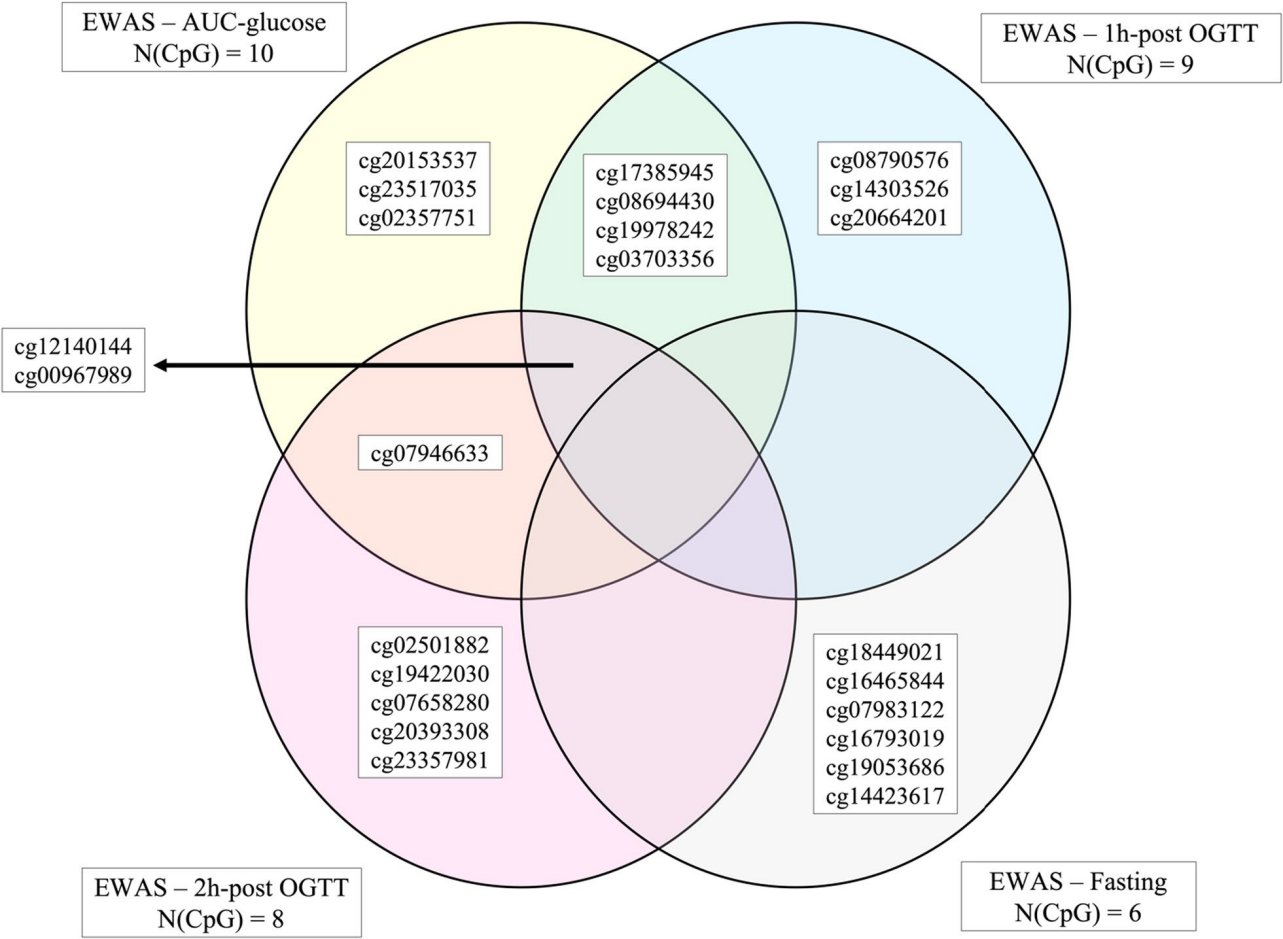
Venn diagram showing common CpGs between maternal AUC_glu_, fasting, 1 h and 2 h post-OGTT glucose levels. All CpG sites shown in the Venn diagram were at least suggestively associated (*P* < 1.0 × 10^–5^) with maternal glucose measurements. Cg00967989 is located at the *FSD1L* gene locus; cg12140144 and cg07946633 are located at the *PRDM16* gene locus

Using LMM, we tested the association between offspring DNAm levels (cord blood and 5-year blood), and maternal fasting glucose, 1 h, and 2 h post-OGTT glucose levels as separate exposures (Additional files 4, 5, and 6). From the 10 CpG sites initially associated with maternal AUC_glu_ (*P*-value ≤ 10^–5^), DNAm levels at cg00967989, located in the *FSD1L* gene, were associated with maternal 1 h-glucose (*β* = − 0.0347, *P* = 6.68 × 10^–8^) and suggestively with the 2 h-glucose (*β* = − 0.0357, *P* = 2.80 × 10^–6^) post-OGTT. DNAm at CpG sites within the *PRDM16* gene associated with maternal AUC_glu_ were suggestively associated with maternal 1 h-glucose (only cg12140144) and 2 h-glucose (both cg12140144 and cg07946633) post-OGTT. In addition, DNAm levels at cg17385945, cg19978242, cg03703356, and cg08694430 located within the *CCDC28A, INPP5F, CKB,* and *RGS3* genes, respectively, were suggestively associated with the 1 h post-OGTT glucose levels (same direction of effect to what we had found in the analyses with maternal AUC_glu_). None of the CpG sites initially associated with maternal AUC_glu_ or with the 1 h- and 2 h-glucose was found associated with fasting glucose levels. These results, along with all CpG sites we found significantly associated with fasting glucose, 1 h and 2 h post-OGTT, are summarized in a Venn diagram (Fig. 4) and presented in Additional files 7, 8, and 9.

## Discussion

In this study, we conducted a longitudinal EWAS based on blood DNAm from birth and 5 years of age in childhood in relation to maternal glycemic response after a 75 g-OGTT conducted during the second trimester of pregnancy. We found CpG sites at which offspring blood DNAm was associated with maternal AUC_glu_ as well as other glycemic indices measured during pregnancy, including 1 h and 2 h post-OGTT glucose levels, but not fasting glucose levels. The different results we obtained with fasting glucose levels could be explained by the different metabolic characteristics observed in the impaired glucose tolerance (IGT) and impaired glucose fasting (IGF) conditions. For example, the insulin resistance state affects different tissues in the two conditions (peripheral in IGT vs. hepatic in IGF) and only IGT subjects show a defect in late-phase insulin secretion in a non-pregnant population [34]. In addition, the two conditions affect different populations that only partially overlap [35], suggesting likely variations in the impact of these complications on potential epigenetic adaptations. Of note, in our previous EWAS of maternal glycemic traits (PACE cohorts meta-analysis), we did not find cord blood DNAm variations that were robustly associated with maternal fasting glucose, while we identified a few CpG sites at which cord blood DNAm was associated with AUCglu and other glycemic indices measured during pregnancy [15]. We identified one CpG site (cg00967989), located in the promotor region of *FSD1L*, at which higher maternal AUC_glu_ was significantly associated with lower values of DNAm at birth and in childhood (after Bonferroni correction for multiple testing). This association was also confirmed with maternal 1 h-glucose and the 2 h-glucose post-OGTT (with a less stringent *P*-value threshold). Our study also reported other CpG sites for which offspring blood DNAm levels were suggestively associated (*P*-value < 10^–5^) with maternal AUC_glu_. Interestingly, two of these were found in the promotor region of *PRDM16* with the same direction of association. We also observed nominal associations between maternal AUC_glu_ and offspring DNAm at individual time-points (cord blood DNAm and blood DNAm at 5 years of age).

The *FSD1L* gene is located on chromosome 9 and encodes for type 2 cystatins. Among other functions, cystatins regulate the activity of endogenous cysteine proteinases which are involved in tumor cell invasion and metastasis [36] potentially via angiogenic properties of *FSD1L* [37]. Genome-wide association studies (GWAS) also reported significant associations between SNPs near its location and age at menarche in females [38], as well as early reproductive traits contributing to fertility in female cattle [39]. However, the exact role of *FSD1L* in humans has not been clearly established yet. Thus, although our results might support its implication in metabolic health programming in the context of prenatal exposure to gestational hyperglycemia, further studies will be needed to better understand the exact role of *FSD1L* in this context.

Our results also suggest lower blood DNAm levels in *PRDM16* promotor at birth and 5 years in the offspring as a response to elevated gestational glycemia. This result at *PRDM16* is consistent in the direction of association with findings by Côté, Gagné-Ouellet et al. who also reported inverse correlations between lower *PRDM16* DNAm levels in the placenta of newborns and higher maternal glycemia [40]. The *PRDM16* gene is located on chromosome 1 and encodes for one of the 17 PRDM protein family members. These proteins contain a PR domain involved in the regulation of transcriptional activity mediated by chromatin histone modifications [41]. *PRDM16* is a transcription factor primarily involved in brown adipose tissue (BAT) differentiation and the transition from white adipose tissue (WAT) to beige adipose tissue (iBAT) [42]. The BAT is enriched in mitochondria whose inner membrane expresses the uncoupling protein 1 (UCP1). UCP1 allows protons to leak across the mitochondrial membrane, which leads to an increase in heat production at the expense of oxidative phosphorylation and adenosine triphosphate (ATP) production [43]. Consistently, BAT and iBAT have been positively associated with leanness and metabolic health in human adults [44]. More specifically, *PRDM16* induces the expression of peroxisome proliferator-activated receptor-y (*PPAR-y*) and *PPAR-y* coactivator-1*α* (*PGC-1α*) with which it forms a complex that regulates the expression of genes associated with lipid catabolism and thermogenesis [45]. Dysregulation of DNAm and gene expression of *PGC-1α* was observed in the placenta of GDM-exposed participants [46]. Studies involving different polymorphisms of *PRDM16* have also shown significant correlations between genetic variants of the gene and the development of obesity in humans [47, 48]. Moreover, Lui et al. [49] demonstrated lower DNAm levels in the promotor region of *PRDM16,* measured in omental adipose tissue (OAT) in individuals with obesity compared with normal-weight subjects, suggesting a role for epigenetic regulation of *PRDM16* in fat mass redistribution of peripheral iBAT to OAT in obesity. However, our findings are based solely on blood DNAm, which may reflect variations in DNAm in other tissues exposed similarly during in utero environmental factors, but we could not biopsy various tissues in healthy newborns for ethical reasons.

Even though computationally intensive, the use of multilevel modeling to analyze trends in DNAm variation is a better strategy to understand the biological relevance of methylation changes over time, versus analyzing their variation at each time-point separately [50]. In our analysis, we identified one signal at *FS1DL* with epigenome-wide significance in the longitudinal analysis and in the analysis of blood DNAm at 5 years. With few exceptions, most of the top signals detected in the longitudinal analysis showed a higher level of statistical significance in this versus the time-point specific analysis, with consistent association estimates observed across analyses. These findings support the advantage of using multilevel modeling over the single time-point analysis to capture CpGs with a time-varying association with the exposure, in this case, maternal hyperglycemia in mid-pregnancy. To our knowledge, this is the first study that has used a longitudinal approach to investigate changes in offspring DNAm associated with maternal hyperglycemia. Previous studies have looked at associations between cord blood DNAm in relation to elevated maternal AUC_glu_ or individual glucose traits measured after an OGTT in pregnancy and have followed up these findings by investigating the cross-sectional association of important baseline markers with cardiometabolic phenotypes in infants and adults [15]. Similarly, the metabolic risk of infants exposed to GDM has been assessed in early childhood (3–10 years of age), identifying epigenetic age acceleration in those born to moms with GDM, which was further associated with cardiometabolic risk factors [51]. Future prospective studies including larger sample sizes and multiple time-points over the life course are warranted to investigate variation in offspring DNAm in response to maternal hyperglycemia in pregnancy and to evaluate the implication of these markers in the offspring’s future health.

## Strengths and limitations

A strength of our study is the prospective cohort design, which allowed collection of samples to evaluate DNAm levels longitudinally from birth to mid-childhood, and their association with maternal glycemia objectively measured during a full 75 g-OGTT in pregnancy. In addition, we measured DNAm using the Illumina MethylationEPIC microarray which provides a coverage of more than 850,000 CpG sites across the human genome. Some limitations are also noteworthy to mention. One CpG site was significantly associated based on a predetermined statistical threshold after correction for multiple testing; however, other findings with suggestive or nominal significance should be interpreted with caution. We investigated DNAm using blood cells; other tissues (liver, beta-cells, adipose, and brain) might be of more relevance for studies of metabolic epigenetic programming related to exposure to maternal hyperglycemia but these tissues are not easily available in healthy children for obvious ethical reasons. We have excluded participants with pre-existing diabetes prior to pregnancy, but 9% of our included population did develop GDM, which may account partially for the observed associations; however, in the past, we have showed that associations between maternal glycemic traits and offspring DNAm markers were stronger in non-GDM participants [15]. Our sample size of participants who had DNAm measurements at both time-points (birth and 5 years) was relatively small (*N* = 194). For this reason, we chose a statistical model (LMM) that combines the two time of measurements, which improves power to detect associations. However, we cannot exclude that other associations with smaller effect sizes were not detected. Finally, our study only included participants of European descent, which limits the application of our results to other races and ethnicities.

## Conclusions

We found associations between maternal hyperglycemia in pregnancy and offspring blood DNAm levels measured at birth and at 5 years. The association was significant after accounting for multiple testing at a CpG site near the *FSD1L* gene. Notably, our study also reports two CpG sites, located in the promotor region of *PRMD16*—a gene implicated in adipose tissue regulation*,* and that we had previously observed associations between maternal hyperglycemia and placenta DNAm. We also confirmed some of our suggestive associations with other maternal glycemic traits, predominantly with 1 h-glucose and 2 h-glucose post-OGTT. Overall, our study provides results supporting that maternal hyperglycemia might be implicated in offspring epigenetic programming which may last until 5 years after birth.

## Supplementary Information

**Additional file 1**: Genomic inflation factor or lambda for each LMM and linear regression models testing associations between maternal hyperglycemia outcomes and DNAm in cord blood and/or in blood at 5 years of age; Table presenting lambda for each LMM and linear regression models.

**Additional file 2**: Differentially methylated regions based on results from LMM testing associations between maternal AUCglu and DNAm measured in cord blood and blood at 5 years of age, using the DMRff method; Table presenting the differentially methylated regions, including their chromosome number, genomic position, number of CpGin the region, estimate value, standard error, *P*-value and adjusted *P*-value, based on results from LMM testing the association between maternal AUCglu and DNAm measured in cord blood at 5 years of age.

**Additional file 3**: CpG sites identified based on LMM or linear regression models testing associations between maternal AUCglu and DNAm in cord blood and/or in blood at 5 years of age; Table presenting CpG sites, including their chromosome number, genomic position, associated gene and regression beta and p-value, identified at suggestive *P* < 10^−5^ based on LMM or linear regression models testing association between maternal AUCglu and DNAm in cord blood and/or in blood at 5 years of age.

**Additional file 4**: Epigenome-wide association plot for maternal fasting glucose levels; Figure of the epigenome-wide association plot presenting CpG sites from the LMM associated with maternal fasting glucose.

**Additional file 5**: Epigenome-wide association plot for maternal 1h-post-OGTT glucose levels; Figure of epigenome-wide association plot presenting CpG sites from the LMM associated with maternal 1h-post-OGTT glucose levels.

**Additional file 6**: Epigenome-wide association plot for maternal 2h-post-OGTT glucose levels; Figure of epigenome-wide association plot presenting CpG sites from the LMM associated with maternal 2h-post-OGTT glucose levels.

**Additional file 7**: CpG sites identifiedin linear mixed models testing associations between maternal fasting glucose and DNAm measured in cord blood and blood at 5 years of age; Table presenting CpG sites, including their chromosome number, genomic position, and associated gene, identified at suggestive *P* < 10^−5^ in linear mixed models testing associations between maternal fasting glucose and DNAm measured in cord blood and blood at 5 years of age.

**Additional file 8**: CpG sites identified in linear mixed models testing associations between maternal 1h-glucose post-OGTT and DNAm measured in cord blood and blood at 5 years of age; Table presenting CpG sites, including their chromosome number, genomic position, and associated gene, identified at suggestive *P* < 10^−5^ in linear mixed models testing associations between maternal 1h post-OGTT glucose levels and DNAm measured in cord blood and blood at 5 years of age.

**Additional file 9**: CpG sites identified in linear mixed models testing associations between maternal 2h-glucose post-OGTT and DNAm measured in cord blood and blood at 5 years of age; Table presenting CpG sites, including their chromosome number, genomic position, and associated gene, identified at suggestive *P* < 10^−5^ in linear mixed models testing associations between maternal 2h post-OGTT glucose levels and DNAm measured in cord blood and blood at 5 years of age.

## Data Availability

The datasets used and/or analyzed during the current study are available from the corresponding author upon reasonable request.

## Abbreviations

ATP: Adenosine triphosphate
AUC_glu_: Area under the curve for glucose
BAT: Brown adipose tissue
BMI: Body mass index
CHUS: Centre hospitalier universitaire de Sherbrooke
CpG: Cytosine–phosphate–guanine
Chr: Chromosome
DNAm: DNA methylation
DOHaD: Developmental origin of health and disease hypothesis
EWAS: Epigenome-wide association study
FG: Fasting glucose
Gen3G: Genetics of glucose regulation in gestation and growth cohort
GCT: 50-G glucose challenge test
GDM: Gestational diabetes mellitus
GWAS: Genome-wide association study
iBAT: Beige adipose tissue
LMM: Linear mixed model
MAF: Minor allele frequency
nRBC: Nucleated red blood cells
OGTT: Oral glucose tolerance test
OAT: Omental adipose tissue
*PGC-1**α*: PPAR-y coactivator-1α
*PPAR-y*: Peroxisome proliferator-activated receptor-y
QC: Quality control
RCP: Regression on correlated probes
SD: Standard deviation
SNP: Single-nucleotide polymorphism
UCP1: Uncoupling protein 1
WAT: White adipose tissue
