# Supplementary material for: Maternal glycemia in pregnancy is longitudinally associated with blood DNAm variation at the FSD1L gene from birth to 5 years of age
Source: Clin Epigenetics. 2023 Jun 29;15:107. doi: 10.1186/s13148-023-01524-7 (PMC10308691; doi:10.1186/s13148-023-01524-7)
Supplement: Supplementary file 1 — Additional file 1: Genomic inflation factor or lambda for each LMM and linear regression models testing associations between maternal hyperglycemia outcomes and DNAm in cord blood and/or in blood at 5 years of age; Table presenting lambda for each LMM and linear regression models. [file 13148_2023_1524_MOESM1_ESM.docx]

**Additional file 1.** Genomic inflation factor or lambda (λ) for each LMM and linear regression models testing associations between maternal hyperglycemia outcomes and DNAm in cord blood and/or in blood at 5 years of age.

| Models | Maternal hyperglycemia outcomes | | | |
| --- | --- | --- | --- | --- |
|  | AUC_glu_ | FG | 1h post OGTT | 2h post OGTT |
| ^a^Random Intercept Model | 1.27 | 0.94 | 1.21 | 1.21 |
| ^b^Cord blood | 1.13 | 0.97 | 1.02 | 1.23 |
| ^b^5 years | 1.09 | 0.94 | 1.14 | 0.94 |

Note: ^a^Linear mixed models adjusted for maternal age, gravidity, smoking status, child sex, BMI at first trimester of pregnancy and the binary variable for time-point. ^b^Linear models adjusted for maternal age, gravidity, smoking status, child sex and BMI at first trimester of pregnancy. Abbreviations: AUC_glu_, Area Under the Curve of glucose; FG, Fasting glucose; LMM, Linear Mixed Model; OGTT, Oral Glucose Tolerance Test.
