# Supplementary material for: Maternal glycemia in pregnancy is longitudinally associated with blood DNAm variation at the FSD1L gene from birth to 5 years of age
Source: Clin Epigenetics. 2023 Jun 29;15:107. doi: 10.1186/s13148-023-01524-7 (PMC10308691; doi:10.1186/s13148-023-01524-7)
Supplement: Supplementary file 2 — Additional file 2: Differentially methylated regions based on results from LMM testing associations between maternal AUCglu and DNAm measured in cord blood and blood at 5 years of age, using the DMRff method; Table presenting the differentially methylated regions, including their chromosome number, genomic position, number of CpGin the region, estimate value, standard error, P-value and adjusted P-value, based on results from LMM testing the association between maternal AUCglu and DNAm measured in cord blood at 5 years of age. [file 13148_2023_1524_MOESM2_ESM.docx]

**Additional file 2.** Differentially methylated regions based on results from LMM testing associations between maternal AUC_glu_ and DNAm measured in cord blood and blood at 5 years of age, using the DMRff method.

| Chr | Start | End | n | Estimate | SE | p-value | Adjusted p-value |
| --- | --- | --- | --- | --- | --- | --- | --- |
| 1 | 2984245 | 2984275 | 2 | -0.0900 | 0.0164 | 4.38e^-08^ | 0.035 |
| 9 | 108210147 | 108210147 | 1 | -0.0992 | 0.0179 | 2.85e^-08^ | 0.023 |

Note: Abbreviations: Chr, Chromosome; n, number of CpG(s) in the region; se, standard error.
