# Supplementary material for: Maternal glycemia in pregnancy is longitudinally associated with blood DNAm variation at the FSD1L gene from birth to 5 years of age
Source: Clin Epigenetics. 2023 Jun 29;15:107. doi: 10.1186/s13148-023-01524-7 (PMC10308691; doi:10.1186/s13148-023-01524-7)
Supplement: Supplementary file 3 — Additional file 3: CpG sites identified based on LMM or linear regression models testing associations between maternal AUCglu and DNAm in cord blood and/or in blood at 5 years of age; Table presenting CpG sites, including their chromosome number, genomic position, associated gene and regression beta and p-value, identified at suggestive P < 10−5 based on LMM or linear regression models testing association between maternal AUCglu and DNAm in cord blood and/or in blood at 5 years of age. [file 13148_2023_1524_MOESM3_ESM.docx]

**Additional table 3.** CpG sites identified (suggestive *P*<10^-5^) based on LMM or linear regression models testing associations between maternal AUC_glu_ and DNAm in cord blood and/or in blood at 5 years of age.

| CpGs | Chr | Position | Gene | ^a^Random Intercept Model | ^b^Cord blood | ^b^5 years |
| --- | --- | --- | --- | --- | --- | --- |
| cg07946633 | 1 | 2984245 | *PRDM16* | β: -0.0206  p: 2.24 x10^-06^ | β: -0.0193  p: 5.36 x10^-04^ | β: -0.0197  p: 1.17 x10^-04^ |
| cg12140144 | 1 | 2984275 | *PRDM16* | β: -0.0251  p: 4.37 x10^-07^ | β: -0.0315  p: 2.26 x10^-06^ | β: -0.0148  p: 0.023 |
| cg20153537 | 3 | 193917170 | *Gencode: LINC02036, UCSC: HES1* | β: -0.0223  p: 2.70 x10^-06^ | β: -0.0252  p: 2.79 x10^-04^ | β: -0.0184  p: 5.67 x10^-04^ |
| cg23517035 | 5 | 133971253 | *SAR1B* | β: 0.0212  p: 8.68 x10^-06^ | β: 0.0236  p: 1.82 x10^-04^ | β: 0.0180  p: 0.011 |
| cg17385945 | 6 | 139095355 | *CCDC28A* | β: -0.0257  p: 9.93 x10^-06^ | β: -0.0312  p: 2.81 x10^-05^ | β: -0.0172  p: 0.061 |
| cg00967989 | 9 | 108210147 | *FSD1L* | **β: -00267**  **p: 2.13 x10^-08^** | β: -0.0152  p: 0.014 | **β: -0.0434**  **p: 4.35 x10^-09^** |
| cg08694430 | 9 | 116420234 | *Gene code: RP11-18B16.2,*  *UCSC: RGS3* | β: 0.0324  p: 1.33 x10^-06^ | β: 0.0329  p: 5.48 x10^-05^ | β: 0.0312  p: 6.60 x10^-04^ |
| cg19978242 | 10 | 121578846 | *INPP5F* | β: -0.0322  p: 4.85 x10^-06^ | β: -0.0314  p: 1.43 x10^-04^ | β: -0.0284  p: 5.33 x10^-03^ |
| cg03703356 | 14 | 103989368 | *CKB* | β: -0.0217  p: 4.25 x10^-07^ | β: -0.0203  p: 3.28 x10^-04^ | β: -0.0211  p: 8.77 x10^-05^ |
| cg02357751 | 22 | 19710880 | *GP1B; SEPT5* | β: -0.0368  p: 7.71 x10^-06^ | β: -0.0194  p: 2.19 x10^-03^ | β: -0.0568  p: 7.21 x10^-04^ |
| cg04858462 | 1 | 26056788 | *MAN1C1* | β: -0.0222  p: 8.27 x10^-05^ | β: -0.0346  p: 6.16 x10^-06^ | β: -0.0043  p: 0.577 |
| cg11358776 | 6 | 114228439 | *FLJ34503* | β: -0.0266  p: 3.65 x10^-05^ | β: -0.0407  p: 3.36 x10^-06^ | β: -0.0045  p: 0.627 |
| cg22587703 | 15 | 40337505 | *SRP14-AS1, or SRP14* | β: -0.0175  p: 4.51 x10^-03^ | β: -0.0355  p: 9.97 x10^-06^ | β: 0.0079  p: 0.410 |
| cg03610266 | 16 | 31471383 | *ARMC5* | β: -0.0209  p: 2.98 x10^-05^ | β: -0.0302  p: 2.70 x10^-06^ | β: -0.0072  p: 0.357 |
| cg04098547 | 16 | 48125057 | *ABCC12* | β: 0.0317  p: 2.12 x10^-03^ | β: 0.0484  p: 5.50 x10^-06^ | β: 0.0097  p: 0.567 |
| cg05413451 | 1 | 57045111 | *PPAP2B* | β: -0.0107  P: 0.014 | β: 0.0012  p: 0.822 | β: -0.0281  p: 8.60 x10^-06^ |
| cg27304328 | 1 | 160519425 | *CD84* | β: 0.0117  p: 3.18x10^-03^ | β: 0.0030  p: 0.578 | β: 0.0220  p: 2.67 x10^-06^ |
| cg12964770 | 2 | 64710206 | *Gene code: LINC01805, UCSC: LGALSL* | β: 0.0128  p: 1.08 x10^-04^ | β: 0.0058  p: 0.181 | β: 0.0226  p: 4.23 x10^-06^ |
| cg19023320 | 3 | 13033587 | *IQSEC1* | β: 0.0101  p: 0.024 | β: -0.0007  p: 0.907 | β: 0.0248  p: 9.95 x10^-06^ |
| cg20997792 | 6 | 29595491 | *GABBR1* | β: -0.0176  p: 1.89 x10^-04^ | β: -0.0082  p: 0.142 | β: -0.0325  p: 9.71 x10^-06^ |
| cg00171729 | 6 | 52416856 | *TRAM2* | β: -0.0129  p: 0.013 | β: 0.0007  p: 0.920 | β: -0.0319  p: 3.41 x10^-06^ |
| cg13860612 | 9 | 136787003 | *VAV2* | β: 0.0142  p: 0.020 | β: -0.0064  p: 0.406 | β: 0.0453  p: 6.24 x10^-07^ |
| cg06731125 | 10 | 21462653 | *NEBL* | β: -0.0184  p: 3.15 x10^-03^ | β: -0.0010  p: 0.901 | β: -0.0449  p: 2.18 x10^-06^ |
| cg17539315 | 12 | 50794919 | *LARP4* | β: -0.0120  p: 0.011 | β: -0.0025  p: 0.704 | β: -0.0255  p: 4.53 x10-^06^ |
| cg09377512 | 17 | 11786949 | *DNAH9* | β: -0.0451  p: 6.37 x10^-05^ | β: -0.0281  p: 0.033 | β: -0.0749  p: 7.01 x10^-07^ |
| cg01774813 | 21 | 36689229 | *RUNX1* | β: 0.0144  p: 1.36 x10-^03^ | β: 0.0002  p: 0.966 | β: 0.0355  p: 2.60 x10^-06^ |

Note: ^a^Model adjusted for maternal age, gravidity, smoking status, child sex, BMI at first trimester of pregnancy and the binary variable for time-point. ^b^Models adjusted for maternal age, gravidity, smoking status, child sex and BMI at first trimester of pregnancy. Significant results with p-value<6.9 x10^-8^ are in bold. Abbreviations: Chr, Chromosome; CpG, Cytosine-phosphate-Guanine.
