# Supplementary material for: Maternal glycemia in pregnancy is longitudinally associated with blood DNAm variation at the FSD1L gene from birth to 5 years of age
Source: Clin Epigenetics. 2023 Jun 29;15:107. doi: 10.1186/s13148-023-01524-7 (PMC10308691; doi:10.1186/s13148-023-01524-7)
Supplement: Supplementary file 6 — Additional file 6: Epigenome-wide association plot for maternal 2h-post-OGTT glucose levels; Figure of epigenome-wide association plot presenting CpG sites from the LMM associated with maternal 2h-post-OGTT glucose levels. [file 13148_2023_1524_MOESM6_ESM.docx]

**Additional file 6.** Epigenome-wide association plot (-log10 p-values) for maternal 2h-post OGTT glucose levels.

**
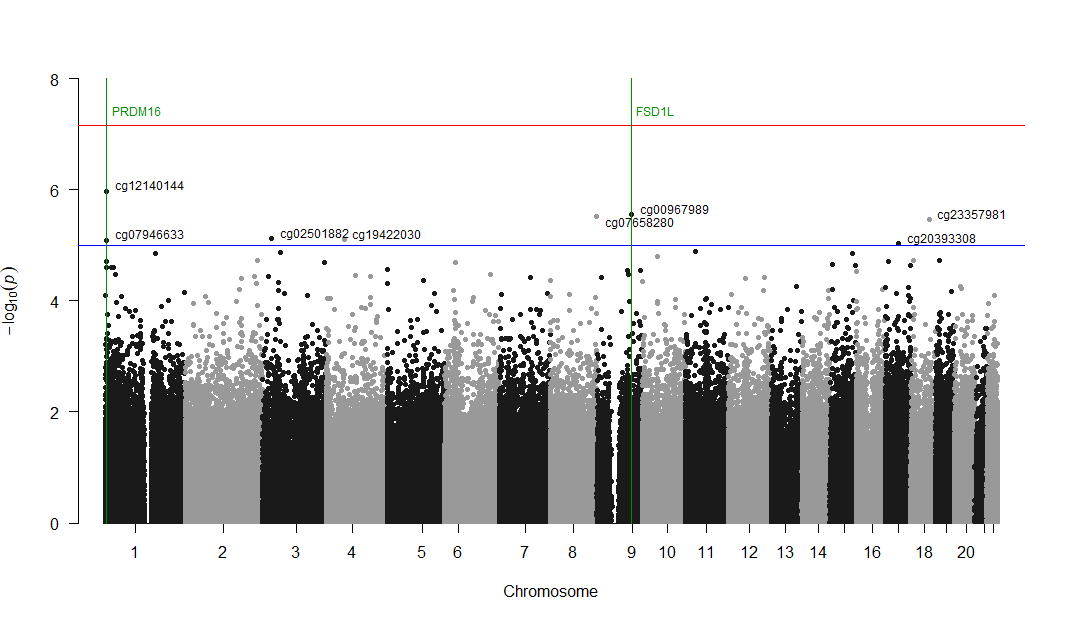
**

Manhattan plot for the EWAS of maternal 2h post-75g-OGTT glucose levels with DNAm measured longitudinally at birth and 5 years of age (red line: Bonferroni threshold= p-value<6.9 x10^-8^; blue line: Suggestive threshold= *P*<1.0 x10^-5^).
