# Supplementary material for: Maternal glycemia in pregnancy is longitudinally associated with blood DNAm variation at the FSD1L gene from birth to 5 years of age
Source: Clin Epigenetics. 2023 Jun 29;15:107. doi: 10.1186/s13148-023-01524-7 (PMC10308691; doi:10.1186/s13148-023-01524-7)
Supplement: Supplementary file 7 — Additional file 7: CpG sites identifiedin linear mixed models testing associations between maternal fasting glucose and DNAm measured in cord blood and blood at 5 years of age; Table presenting CpG sites, including their chromosome number, genomic position, and associated gene, identified at suggestive P < 10−5 in linear mixed models testing associations between maternal fasting glucose and DNAm measured in cord blood and blood at 5 years of age. [file 13148_2023_1524_MOESM7_ESM.docx]

**Additional file 7.** CpG sites identified (suggestive *P*<10^-5^) in linear mixed models testing associations between maternal fasting glucose and DNAm measured in cord blood and blood at 5 years of age.

| CpGs | Chr | Position | Gene | FG |
| --- | --- | --- | --- | --- |
| cg18449021 | 5 | 107004816 | *EFNA5* | β: 0.1474  p: 5.83 x10^-06^ |
| cg16465844 | 6 | 13295982 | *LOC100130357* | β: -0.1089  p: 1.62 x10^-06^ |
| cg07983122 | 6 | 24402124 | *MRS2* | β: 0.1308  p: 9.40 x10^-06^ |
| cg16793019 | 6 | 31799404 |  | β: 0.1864  p: 8.91 x10^-06^ |
| cg19053686 | 7 | 43479485 | *HECW1* | β: 0.1527  p: 5.66 x10^-06^ |
| cg14423617 | 14 | 95058208 | *SERPINA5* | β: -0.1444  p: 1.84 x10^-06^ |

Note: Model adjusted for maternal age, gravidity, smoking status, child sex, BMI at first trimester of pregnancy and the binary variable for time-point. Abbreviations: Chr, Chromosome; CpG, Cytosine-phosphate-Guanine; FG, Fasting Glucose.
