# Supplementary material for: Maternal glycemia in pregnancy is longitudinally associated with blood DNAm variation at the FSD1L gene from birth to 5 years of age
Source: Clin Epigenetics. 2023 Jun 29;15:107. doi: 10.1186/s13148-023-01524-7 (PMC10308691; doi:10.1186/s13148-023-01524-7)
Supplement: Supplementary file 8 — Additional file 8: CpG sites identified in linear mixed models testing associations between maternal 1h-glucose post-OGTT and DNAm measured in cord blood and blood at 5 years of age; Table presenting CpG sites, including their chromosome number, genomic position, and associated gene, identified at suggestive P < 10−5 in linear mixed models testing associations between maternal 1h post-OGTT glucose levels and DNAm measured in cord blood and blood at 5 years of age. [file 13148_2023_1524_MOESM8_ESM.docx]

**Additional file 8.** CpG sites identified (suggestive *P*<10^-5^) in linear mixed models testing associations between maternal 1h-glucose post-OGTT and DNAm measured in cord blood and blood at 5 years of age.

| CpGs | Chr | Position | Gene | 1h post OGTT |
| --- | --- | --- | --- | --- |
| cg12140144 | 1 | 2984275 | *PRDM16* | β: -0.0317  p: 2.34 x10^-06^ |
| cg17385945 | 6 | 139095355 | *CCDC28A* | β: -0.0359  p: 4.43 x10^-06^ |
| cg08790576 | 8 | 47846324 | *Gene code: RP11-350F16.2, UCSC: LINC00293 or SPIDR* | β: 0.0390  p: 2.48 x10^-06^ |
| cg00967989 | **9** | **108210147** | ***FSD1L*** | **β: -0.0347**  **p: 6.68 x10^-08^** |
| cg08694430 | 9 | 116420234 | *Gene code: RP11-18B16.2, USCSC: RGS3* | β: 0.0467  p: 2.40 x10^-07^ |
| cg14303526 | 10 | 101609768 | *ABCC2* | β: -0.0338  p: 9.25 x10^-06^ |
| cg19978242 | 10 | 121578846 | *INPP5F* | β: -0.0429  p: 6.74 x10^-06^ |
| cg20664201 | 12 | 57849270 | *INHBE* | β: 0.0181  p: 8.63 x10^-06^ |
| cg03703356 | 14 | 103989368 | *CKB* | β: -0.0288  p: 7.03 x10^-07^ |

Note: Model adjusted for maternal age, gravidity, smoking status, child sex, BMI at first trimester of pregnancy and the binary variable for time-point. Significant results with p-value<6.9 x10^-8^ are in bold. Abbreviations: Chr, Chromosome; CpG, Cytosine-phosphate-Guanine; OGTT, 75-gram fasting Oral Glucose Tolerance Test.
