# Supplementary material for: Maternal glycemia in pregnancy is longitudinally associated with blood DNAm variation at the FSD1L gene from birth to 5 years of age
Source: Clin Epigenetics. 2023 Jun 29;15:107. doi: 10.1186/s13148-023-01524-7 (PMC10308691; doi:10.1186/s13148-023-01524-7)
Supplement: Supplementary file 9 — Additional file 9: CpG sites identified in linear mixed models testing associations between maternal 2h-glucose post-OGTT and DNAm measured in cord blood and blood at 5 years of age; Table presenting CpG sites, including their chromosome number, genomic position, and associated gene, identified at suggestive P < 10−5 in linear mixed models testing associations between maternal 2h post-OGTT glucose levels and DNAm measured in cord blood and blood at 5 years of age. [file 13148_2023_1524_MOESM9_ESM.docx]

**Supplementary file 9.** CpG sites identified (suggestive *P*<10^-5^) in linear mixed models testing associations between maternal 2h-glucose post-OGTT and DNAm measured in cord blood and blood at 5 years of age.

| CpGs | Chr | Position | Gene | 2h post OGTT |
| --- | --- | --- | --- | --- |
| cg07946633 | 1 | 2984245 | *PRDM16* | β: -0.0309  p: 8.04 x10^-06^ |
| cg12140144 | 1 | 2984275 | *PRDM16* | β: -0.0386  p: 1.06 x10^-06^ |
| cg02501882 | 3 | 27410970 | *NEK10* | β: -0.0230  p: 7.60 x10^-06^ |
| cg19422030 | 4 | 56501718 | *NMU* | β: -0.0387  p: 8.00 x10^-06^ |
| cg07658280 | 8 | 145728490 | *GPT* | β: 0.0225  p: 3.05 x10^-06^ |
| cg00967989 | 9 | 108210147 | *FSD1L* | β: -0.0357  p: 2.80 x10^-06^ |
| cg20393308 | 17 | 43728044 | *MGC57346-CRHR1* | β: 0.0619  p: 9.27 x10^-06^ |
| cg23357981 | 18 | 56887785 | *GRP* | β: -0.0245  p: 3.46 x10^-06^ |

Note: Model adjusted for maternal age, gravidity, smoking status, child sex, BMI at first trimester of pregnancy and the binary variable for time-point. Abbreviations: Chr, Chromosome; CpG, Cytosine-phosphate-Guanine; OGTT, 75-gram fasting Oral Glucose Tolerance Test.
